# Behind the Scenes of Anthocyanins—From the Health Benefits to Potential Applications in Food, Pharmaceutical and Cosmetic Fields

**DOI:** 10.3390/nu14235133

**Published:** 2022-12-02

**Authors:** José S. Câmara, Monica Locatelli, Jorge A. M. Pereira, Hélder Oliveira, Marco Arlorio, Iva Fernandes, Rosa Perestrelo, Victor Freitas, Matteo Bordiga

**Affiliations:** 1 CQM—Centro de Química da Madeira, Natural Products Research Group, Campus Universitário da Penteada, Universidade da Madeira, 9020-105 Funchal, Portugal; 2 Departamento de Química, Faculdade de Ciências Exatas e Engenharia, Campus da Penteada, Universidade da Madeira, 9020-105 Funchal, Portugal; 3 Department of Pharmaceutical Sciences, Università degli Studi del Piemonte Orientale “A. Avogadro”, Largo Donegani 2, 28100 Novara, Italy; 4 LAQV, REQUIMTE, Departamento de Química e Bioquímica, Faculdade de Ciências, Universidade do Porto, Rua do Campo Alegre s/n, 4169-007 Porto, Portugal

**Keywords:** anthocyanins (ACNs), occurrence, health benefits, bioactivity, food applications, pharmaceutical applications, cosmetic applications

## Abstract

Anthocyanins are widespread and biologically active water-soluble phenolic pigments responsible for a wide range of vivid colours, from red (acidic conditions) to purplish blue (basic conditions), present in fruits, vegetables, and coloured grains. The pigments’ stability and colours are influenced mainly by pH but also by structure, temperature, and light. The colour-stabilizing mechanisms of plants are determined by inter- and intramolecular co-pigmentation and metal complexation, driven by van der Waals, π–π stacking, hydrogen bonding, and metal-ligand interactions. This group of flavonoids is well-known to have potent anti-inflammatory and antioxidant effects, which explains the biological effects associated with them. Therefore, this review provides an overview of the role of anthocyanins as natural colorants, showing they are less harmful than conventional colorants, with several technological potential applications in different industrial fields, namely in the textile and food industries, as well as in the development of photosensitizers for dye-sensitized solar cells, as new photosensitizers in photodynamic therapy, pharmaceuticals, and in the cosmetic industry, mainly on the formulation of skin care formulations, sunscreen filters, nail colorants, skin & hair cleansing products, amongst others. In addition, we will unveil some of the latest studies about the health benefits of anthocyanins, mainly focusing on the protection against the most prevalent human diseases mediated by oxidative stress, namely cardiovascular and neurodegenerative diseases, cancer, and diabetes. The contribution of anthocyanins to visual health is also very relevant and will be briefly explored.

## 1. Anthocyanins Classification, Chemical Properties and Biosynthesis

The benefits of consuming medicinal herbs, fruits, and vegetables on a regular basis for human health have gained widespread acceptance in recent years. This is mostly due to their composition, which is rich in several non-nutrient bioactive chemicals, such as phenolics, which have long been known for their ability to influence various processes and pathways in the human body, such as regulating glucose levels and increasing anti-inflammatory, antioxidant, anticancer, anti-mutagenic, and neuroprotective properties [1]. Among the phenolic compounds, one of the polyphenols class’s most extensively studied members is the anthocyanins (ACNs), representing an interesting class of water-soluble compounds (pigments). One reason for this is that they offer a viable alternative to the most used synthetic food dyes, which can produce hazardous consequences for human health, and are thus becoming increasingly significant to the food business and consumers. The flavonoid pathway is the one responsible for the synthesis of anthocyanins. Many fruits, vegetables, and edible flowers, as well as their derivatives such as juices, red wines, and tea, exhibit intense red, orange, violet, and blue colours [2]. They are the primary sources of these colours. In addition, they have drawn a lot of interest because of their nutritional value, pharmacokinetic profile, pharmacological mechanisms, and health-promoting qualities [3].

These molecules are useful substances that can boost antioxidant defences, reduce the effects of chronic inflammation, free radicals, and mutation risk, as well as slow or even stop the onset and progression of many chronic non-communicable and degenerative diseases. Anthocyanins are natural colorants that are less hazardous than synthetic ones and have a variety of technological potential applications in a variety of industrial domains, including the food and textile industries. The goal of this comprehensive review was to evaluate and clarify these applications. We also discussed some of the most recent research on the health advantages of anthocyanins, with a particular emphasis on the prevention of the most common oxidative stress-related human disorders, such as cancer, diabetes, and cardiovascular and neurological diseases.

### 1.1. Classification and Chemical Properties

The ACNs are thought to be the biggest, most fascinating, and intriguing group of plant-based pigments used by humans since the dawn of time as colourants agents for foods, drinks, and clothing, as well as for baits, armour, phytopharmaceuticals, colours for drawings and cave art, and celebrations [1,2,3]. The Greek words for anthocyanin (ACN) are “*anthos*”, which means flower, and “*kyaneos*”, which means dark blue tint. Due to their multicoloured appearances in many visible plant components, such as flowers, fruits, leaves, tubers, and roots, they are found in plant cell vacuoles. As a result, they have attracted attention in a variety of human activities, including those involving medicine. In addition to attracting insects and other animals, ACNs are also attractive to people. They are also indirect carriers of plant species spread, conservation, and natural balances [4].

Nearly one-third of all flavonoids are ACNs, which are structurally polyphenolic and water-soluble. They are primarily found in the plums, cherries, and berries of various plants, and they have developed various shades of purple, red, violet, pink, and blue, indicating the apparent presence of this class of compounds in nature. One of the main contributions is the plant family *Rosaceae*, which also includes *Berberidaceae*, *Eleaocarpaceae*, *Myrtaceae*, and *Solanaceae* [5,6].

The ACNs serve as an advantageous barrier that protects plants from UV-B radiation and excessive light input [7]. As antioxidant plant components, ACNs protect plants from a variety of abiotic challenges, such as drought and high saline levels, as well as heat and light impacts.

Additionally, ACNs play a role in the transfer of monosaccharides, camouflage, osmotic balance, and senescence [7]. As a result, ACNs serve a crucial role in preventing DNA damage to plants and shielding their photosynthetic mechanism from excessive light radiation flux [8]. In addition, ACNs assist control plant haemostasis and offer protection from drought, heat, cold, and water stress [9]. Additionally, ACNs’ function in defending plants from various biotic stressors, such as microbial and insect attacks, has been documented [10,11]. The ACNs found in plants differ depending on the plant’s species and variety, the predominant environmental conditions, the stage of growth the plant is in, and how the plant’s products are stored [12]. ACNs are spread throughout numerous plant parts, especially in the Autumn, and are also collected over time in plant vacuoles, reaching their peak at the time when the fruits are ripe [13,14].

**Figure 1:**
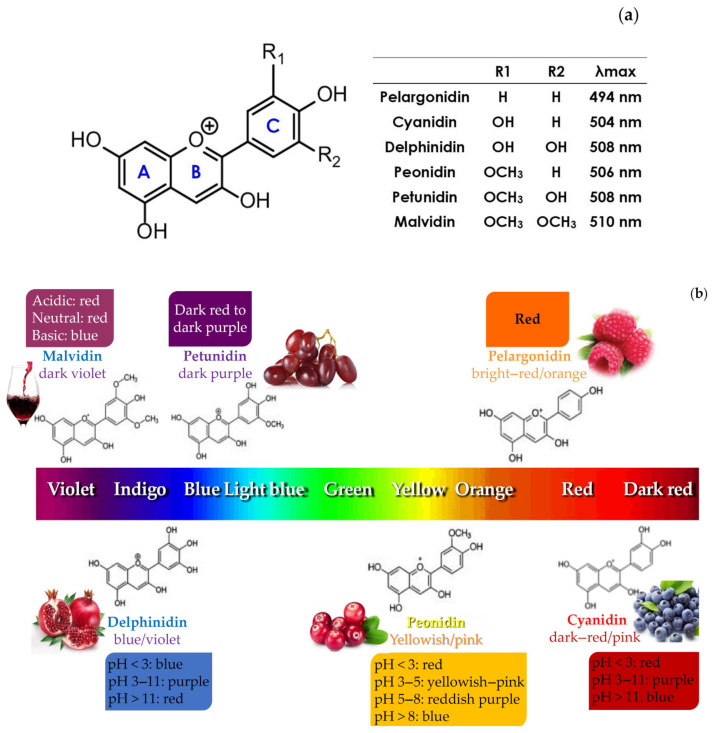
(**a**) Basic skeleton and major ACNs in plants, with respective maximum wavelength; (**b**) Spectral absorbance ranges for the most-abundant anthocyanins and respective colour at different pH values.

The ACNs have been classed as belonging to the C6-C3-C6 molecular framework and are structurally a part of the flavonoid series of plant compounds. The anthocyanin products are identified as the sugar-bonded counterparts (glycosides) of the anthocyanidins (aglycones) analogues [15], where the flavylium cation’s fifteen-carbon atom-based framework skeleton is structured in three rings indicated as A, B, and C, and is a component of the ACN compounds’ basic skeleton (Figure 1). The three-ring structure of ACN compounds typically has many hydroxylation patterns, particularly at the C-3 position of ring C, the C-5, C-6, and C-7 positions of ring A, and the C-3, C-4, and C-5 positions of ring B [16]. Through the creation of an acetal linkage with one or more of the hydroxyl groups discussed earlier, the sugar moieties are joined to the anthocyanidins’ structural components. By glycosylating the 3-OH group to create the 3-*O*-glucoside derivatives, such as cyanidin-3-*O*-glucoside (Cy3glu) and peonidin-3-*O*-glucoside, the most prevalent and plentiful ACN glycosides are created [17].

The extremely resonant electrons around the flavylium ion structure are responsible for the ACN compounds’ ability to give plants varied colours [18,19,20]. In addition, a wide range of ACN hydroxylation patterns around the three rings (rings A, B, and C) of the compounds, as well as the kinds and locations of glycosylation and carboxylates connected to the sugar moieties, all contribute to the diversification of the recognized ACN compounds’ hues [15,20]. Over 700 ACN compounds from various plant sources have been found and grouped under various chemical classes based on structural variations. The 27 various varieties of anthocyanidin, the aglycone molecular framework that creates the diverse ACNs due to their structural variances, have been used to identify the ACNs.

It’s interesting to note that ACNs are rarely found in nature as aglycones; instead, almost all of them exist as glycosides, of which about half have acylated structures. ACNs colours’ chemistry includes pH-based modulations [20,21]. When hydrated, the quinonoid base (pH 8–10) changes to pH 2 of the flavylium cation, which has a reddish-to-orange colour. This generates the carbinol pseudo-base (pH range 3–6), which is then followed by the chalcone pseudo-base, which is also colourless. However, in addition to pH and ACN structure, other elements such as oxygen, moisture, metals, enzymes, light exposure, and temperature significantly affect the colour and durability of these products [20,22]. For example, the presence of hydroxyl and methoxy groups reduces ACN stability.

Additionally, the co-conjugates of tannin and ACN boost colour stability at lower pH [23]. The ACN aglycone’s co-pigmentation with flavonoids, which is made possible by the presence of metallic ions, also stabilizes the colour. The glycosylation and acetylation of anthocyanidins play a major role in the maintenance of the ACN’s colour [20,24]. For the reason ACNs are extremely delicate substances, other substances including proteins, phenolic acids, and enzymes can cause them to flocculate and change colour [15,25]. The ACN molecules, however, degrade at very high pH levels and lose their colour [26].

### 1.2. Biosynthesis

**Figure 2:**
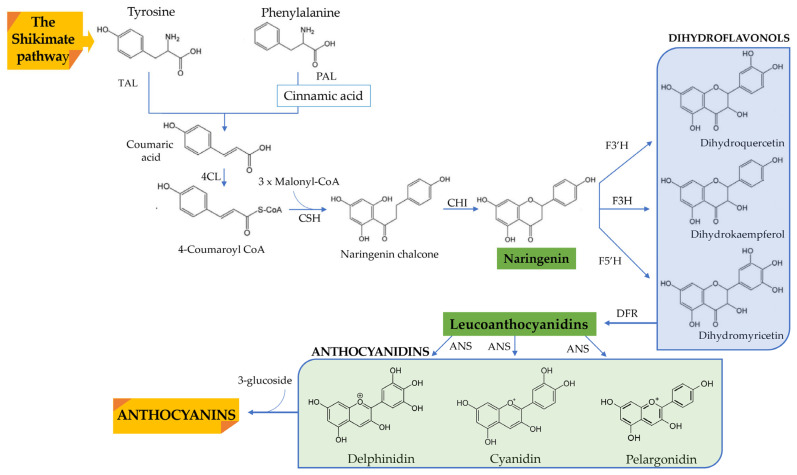
Overview of ACNs biosynthesis pathway (adapted from Zhang et al. [33], Liu et. al. [34] and Cruz, Basilio, Mateus, de Freitas and Pina [20]). 4CL: 4-coumaroyl-CoA ligase; ANS: anthocyanidin synthase; CHI: chalcone isomerase; CSH: chalcone synthase; DFR: dihydroflavonol 4-reductase; F3H: flavanone 3- hydroxylase; F3′H: flavonoid 3′-hydroxylase; F5′H: flavonoid 5′-hydroxylase; PAL: phenylalanine ammonia lyase.

ACNs’ synthesis process has been well studied in both the model plant *Arabidopsis thaliana* and several different crops, and it seems to be highly conserved. An essential branch of the phenylpropanoid route, the ACN biosynthesis pathway shares certain beginning steps with other flavonoids’ biosynthetic pathways, including those for flavones and flavonones [27,28]. It begins with phenylalanine, which phenylalanine ammonia-lyase (PAL) converts into cinnamic acid (Figure 2).

Cinnamate-4-hydroxylase converts the cinnamic acid into coumaric acid, which is then transformed into 4-coumaroil CoA by the 4-coumaroil CoA ligase. Cinnamate-4-hydroxylase converts the cinnamic acid into coumaric acid, which is then transformed into 4-coumaroil CoA by the 4-coumaroil CoA ligase. Chalcone synthase produces naringenin chalcone after 4-coumaroil CoA condenses with malonyl CoA. It was then transformed into naringenin by the enzymes chalcone isomerase (CHI) and dihydroflavonols (dihydrokaempferol and dihydroquercetin) from the flavanone 3-hydroxylase (F3H) and flavonoid 30-hydroxylase (F30H), respectively.

Leucocyanidins, cyanidins, and ACNs are produced in the biosynthetic pathway’s last steps by the enzymes dihydroflavonol reductase (DFR), anthocyanidin synthase (ANS), and UDP-glucose:flavonoid-3-*O*-glycosyltransferase (UFGT), respectively. ACNs are produced in the cytosol by a specific set of transporters, where they are then stored in the vacuole. The transit of ACNs and their vacuolar accumulation in the seed have been linked to the TT12 and AHA10 genes in *Arabidopsis*, for instance [29,30].

It has been demonstrated that TT12, a gene that codes for a membrane protein that is a member of the “multidrug and toxic eux antiporter” family, is particularly important for the accumulation of glycosylated flavan-3-ols and protoanthocyanidins at the vacuolar level. Instead, the acidification of the vacuole is brought on by AHA10, which encodes for a plasma membrane H+-ATPase [31]. Similar characteristics, including transparent heads on the seeds and changes in the ACN accumulation in the vacuole, are seen in both *tt12* and *aha10* mutants. AHA10 is believed to be capable to supply the proton gradient required for the mediated TT12 transport precisely for this reason. In addition, it has recently been shown that “ATP-binding cassette (ABC)” and “H^+^” antiport” are engaged in the vacuolar transport of these molecules in *Arabidopsis* vegetative tissues [32]. These transport mechanisms are also promoted by the intimate contact between ACNs and GSH.

### 1.3. Dietary Sources

#### 1.3.1. Flowers

ACNs are a class of water-soluble flavonoids that give flower petals their appealing red, purple, blue, or pink hue. Due to their powerful antioxidant properties, ACNs play a significant role in addition to serving as colourants [35]. By scavenging free radicals, chelating metals, and controlling antioxidant enzymes, ACNs’ reducing ability allows them to perform antioxidant activity. Due to their capacity to combine with DNA to produce co-pigments, ACNs can protect DNA against lipid peroxidation. They also display antioxidant cellular processes similar to those of other micronutrients and regulate the harm caused by oxidative stress on biomolecules [36].

According to an analysis of the ACN content of edible flowers, cyanidin, delphinidin, pelargonidin, petunidin, peonidin, and malvidin are the ACNs that are most frequently present in flowers [37]. According to research by Zheng et al. [38] on 70 types of edible flowers from China, Cy3glu was the most common ACN in the flowers under study. It was established that variations in colour within the anther, pollen, and perianth of flowers belonging to the same species were caused by the presence and make-up of ACNs. For instance, the dark purple colour of *Tulipa gesneriana* pollens and anthers was caused by the presence of the rare pelargonidin 3-(2‴-acetyl rutinoside) and cyanidin 3-(2‴-acetyl rutinoside), whereas the red colour of perianths was caused by the presence of pelargonidin 3-rutinoside, cyanidin 3-rutinoside, and their derivatives [39]. According to Zhao et al. [40], the pinkish-blue and reddish-purple hues of the petals of *P. suffruticosa* are a result of the presence of pelargonidin and peonidin derivatives.

For instance, the *Catharanthus roseus* cultivar’s pale red colour is caused by the compounds 7-*O*-methylpelargonidin 3-*O*-robinobioside and 7-*O*-methylpelargonidin 3-*O*-galactoside, whereas the reddish-purple colour is caused by the compounds hirsutidin 3-*O*-robinobioside, hirsutidin 3-*O*-robinobioside-5-*O*-galactoside [41].

The blue hue of the *Centaurea cyanus* flower is a result of cyanidin 3-*O*-(6″-succinylglucoside)-5-*O*-glucoside. Delphinidin 3-*O*-(6″-malonylglucoside) and delphinidin 3-*O*-(3″,6″-dimalonylglucoside) are the two ACNs that give transgenic *C. morifolium* flowers their purple-violet hue [42].

The major pigments found in higher plants are flavonoids, carotenoids, and chlorophylls. The last two classes of these are the pigments that give flowers their colours. There’s probably a connection between animal and plant colours in flower colours. Particularly, colour-attracted insects and birds pollinate the flowers and help plant species survive. Contrary to humans, many insects can sense in both the visible and near UV ranges (340–380 nm).

The most significant flower pigments are ACNs, which range in colour from orange to purple to blue and occasionally black. However, betalain pigments particularly express their hues in the order of *Caryophyllales*. Pink, scarlet, crimson, red-purple, and magenta flowers typically contain cyanidin and/or pelargonidin, with or without peonidin, as their ACNs. A few acylated cyanidin glycosides, for instance, are responsible for the crimson flowers of several *Clematis cultivars* (*Ranunculaceae*) [43]. *Pharbitis nil* cultivars (*Convolvulaceae*), Verbena cultivars (*Verbenaceae*), Delphinium cultivars (*Ranunculaceae*), and Gladiolus cultivars (*Iridaceae*) cultivars all have flowers that are red, red-purple, crimson, deep pink, and magenta in hue. Pelargonidin glycosides express these colours. The transgenic *Chrysanthemum morifolium* cultivar’s purple-violet blooms, for instance, contain two malonylated delphinidin 3-*O*-glucosides as ACNs. Delphinidin glycosides are also responsible for the purple and light blue-purple flowers of the Campanula medium cultivar and *Ranunculus asiaticus* (both *Ranunculaceae*).

When ACNs and their C-glycosylflavones coexist, the flower’s colour becomes more bluish (inter-molecular copigmentation). Purple Gladiolus cultivar flowers exhibit intermolecular copigmentation. Malvidin 3,5-di-*O*-glucoside, the main ACN in this case, and the flavonols kaempferol 3-*O*-rutinoside and 3-*O*-[glucosyl-(12)-rhamnoside] are co-pigment substances.

#### 1.3.2. Foods—Vegetables and Fruits

ACNs are present in large quantities in red/blue fruits and vegetables, and their amount in plants varies significantly between species, depending on the cultivar or variety, growing region, climate, farming practices, harvesting period, ripening, seasonal variability, processing and storage, temperature, and light exposure. ACN levels in berries range from about 100 to roughly 700 mg/100 g of fresh product [44,45], with elderberries and chokeberries having the greatest amounts at up to 1.4–1.8 g/100 g. Berries such as strawberries, blueberries, blackberries, blackcurrant, redcurrant, and raspberries are also rich sources of ACNs.

Purple corn, cherries, plums, pomegranates, eggplant, wine, grapes, and red/purple vegetables such as black carrots, red cabbage, and purple cauliflower are other excellent sources of ACNs [44,45]. These foods can have concentrations of as little as a few milligrams to as much as 200–300 mg/100 g of product. More recently, ACNs have been discovered in many berries, including maqui [46,47], myrtle [48,49], and açai [50,51,52], whose production and consumption are both rising significantly.

The most common pigment in plants is cyanidin, which has two hydroxyl groups on the B-ring. Delphinidin, pelargonidin, and peonidin glucosides are the next most prevalent ACNs in edible plants, respectively, after Cy3glu [53]. Methylation induces a red colour and increases stability while hydroxylation produces a blue hue and decreases stability. Glycosylation or acylation are two processes that can change the hydroxyl groups. Both alterations alter the reactivity and polarity of the molecules as well as the physical and chemical characteristics of ACNs.

Up to 70% of one’s daily intake of ACNs comes from fruits, primarily apples, pears, berries, stone fruits, and grapes. Up to 25% of the total intake in Europe may come from wine [54]. Berries are the principal dietary sources in the US and Northern Europe [55,56]. Since ACNs are not regarded as necessary nutrients, there is no set recommended daily consumption; nonetheless, China recently advised a daily intake of 50 mg [57].

It has been estimated that the daily consumption of ACNs in the US is about 12.5 mg/day [45], whereas in Europe the mean intake ranges from 19 to 65 mg/day for males and from 18 to 44 mg/day for women [54]. However, estimating the daily intake of ACNs is difficult and imprecise, mostly because there is insufficient data on the quantities of ACNs in food. According to a study from Australia, the average daily intake of ACNs is around 24 mg [58]; however, in Finland, the daily intake has been calculated to be up to 150 mg [56], with berries being the main source.

To provide a sufficient level of antioxidant and protective chemicals at the plasma level, it may be advantageous to promote the consumption of fresh fruits and vegetables given the health-protective benefits of ACNs. Consuming fruits, and vegetables daily can protect against chronic and degenerative diseases and is a key component of a healthy lifestyle. For example, following a Mediterranean diet, which is high in ACNs-rich foods (fruits, berries, vegetables, beans, and cereals), has been linked to reduced levels of inflammation markers and a lower risk of several diseases, such as obesity, diabetes, cancer, and CVDs [59]. On the other hand, a lack of fruits and vegetables is thought to be the cause of 1.7 million fatalities worldwide, including but not limited to those brought on by ischemic heart disease (11%), stroke (9%), and gastrointestinal cancer (14%). Berries, red wine, vegetables, and other fruits are typically the main sources of ACNs [60]. Elderberries, chokeberries, blueberries, pomegranate, and açai all contain significant amounts of ACNs, with levels exceeding 300 mg of cyanidin 3-glucoside equivalent per 100 g of fresh product [61,62]. The most prevalent ACN glycosides are cyanidin, malvidin, and delphinidin [61,63,64,65]. The primary sources of ACNs are listed in Table 1.

## 2. Significance of ACNs, from Plants to Human Health Benefits

**Figure 3:**
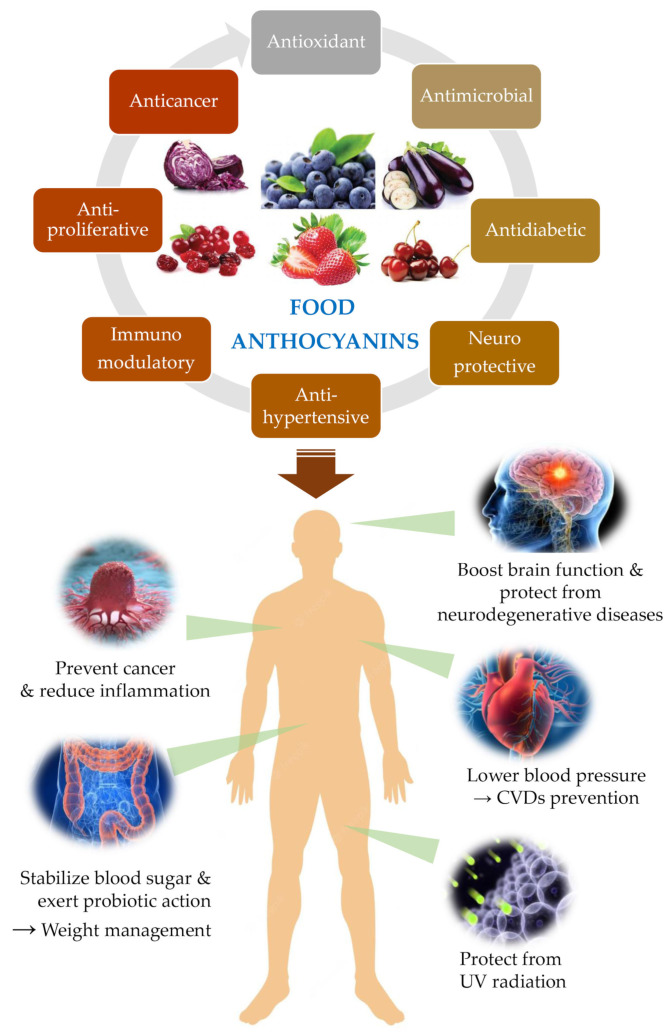
Overview of major human protective effects associated to ACNs consumption reported in the literature.

Plants are organisms not provided with motility organs, and for this reason they are highly exposed to external conditions, namely unfavourable edaphoclimatic parameters such as drought, high-temperature fluctuations, and radiation exposure, salinity, soil nutrients deficiency, among others. To cope with these challenges, plants produce a huge diversity of secondary metabolites that allowed their survival and colonization of the most disperse ecosystems on the planet. But these metabolites also confer protection and advantages to other organisms that include plants in their diets, namely herbivores and mammals (Figure 3).

Overall, the benefits associated with ACNs consumption for the promotion and protection of human health span the most diverse functions and effects in our body as synthetised in Figure 3.

Effectively, there is an overwhelming number of plant secondary metabolites that confer protection to humans, and ACNs are among these bioactive compounds. In this chapter, we will unveil some of the latest studies about the health benefits of ACNs, mainly focusing on the protection against the most prevalent human diseases, namely cardiovascular and neurodegenerative diseases, cancer, and diabetes. The contribution of ACNs to visual health is also very relevant and will be briefly explored (Figure 3).

### 2.1. Cardiovascular Diseases

Cardiovascular diseases (CVDs) are a set of disorders that affect the heart and blood vessels and have earned increasing awareness due to high prevalence and mortality worldwide [89,90,91]. It has been expected that the ratio of CVD-related deaths to worldwide deaths would rise from 28% in 1990 to 31.5% in 2020 [89]. Hypertension, dyslipidemia, atherosclerosis, oxidative stress, inflammation, and enteric dysbacteriosis are the major conditions linked to CVDs [91]. Moreover, the development of CVDs is connected to the production of free radicals from several sources, namely the mitochondrial electron transport chain, angiotensin II-mediated NADPH oxidase system, xanthin oxidase, and inducible nitric oxidase synthase [92]. In this sense, many synthetic drugs have been applied to the treatment of CVDs, but some side effects, namely gastrointestinal reaction, hyperkalemia, and arrhythmias, were also identified [91]. On the other hand, key findings from epidemiological and clinical studies revealed an inverse relationship between CVDs and the intake of some antioxidant natural products (e.g., fruits, vegetables, teas, cereals, nuts) [90,91] (Table 2). Moreover, research reported that consumption of ACN-rich foods (e.g., berries, nuts) [93,94,95], ACN extracts [89,96,97], and purified ACNs as supplements [98,99,100] resulted in beneficial functions in the prevention of CVDs, such as decreasing of low-density lipoprotein-cholesterol (LDL-C), triglycerides, blood pressure, inflammatory and oxidative stress biomarkers, as well as increasing high-density lipoprotein-cholesterol (HDL-C) and improving vascular endothelial functions. Nevertheless, the effective dose of ACN necessary to promote beneficial functions has not been well established.

Regarding the consumption of anthocyanin-rich foods (e.g., berries, nuts), Askari et al. [95] investigated the association between nuts and legume consumption with Framingham risk score and CVD risk factors in older adult men. The results suggest that older adult men with higher consumption of these food products have higher serum levels of HDL-C and are less likely to have high LDL-C serum levels compared with those with lower consumption. However, no association was observed between nuts and legumes with Framingham’s risk score and other CVD risk factors. On the other hand, Nurfaradilla et al. [109] evaluate the effect of *Hibiscus sabdariffa* extract coadministration with the frequently prescribed angiotensin-converting enzyme (ACE) inhibitor captopril on blood pressure and biomarkers of the renin-angiotensin-aldosterone system. The data obtained suggest that *Hibiscus sabdariffa* aqueous extract has potential in the prevention of CVDs, since the plasma renin level, serum angiotensin-converting enzyme (ACE) activity, and plasma angiotensin II level were also significantly reduced. Del Bo’ and collaborators [97] examined the ability of an ACN-rich extract from blueberry, single ACNs (e.g., cyanidin, delphinidin, and malvidin-3-glucoside), and related metabolites (e.g., protocatechuic, gallic and syringic acid) to reduce the inflammation-driven adhesion of monocytes (THP-1) on endothelial cell and secretion of cell adhesion molecules E-selectin and vascular cell adhesion molecule (VCAM-1). The anthocyanin-rich extract and malvidin-3-glucoside (Mv3glu) had an effect at all the concentrations analysed, whereas Cy3glu decreased the adhesion by about 41.8% only at the high concentrations (10 μg/mL).

Previous in vivo studies have demonstrated that ACN supplementation improves anti-oxidative and anti-inflammatory capacity in a dose-response manner in subjects with CVDs. In this sense, Zhang, and collaborators [101] performed a randomized controlled trial to evaluate the dose-response effect of ACNs supplementation on oxidative and inflammatory response in individuals with dyslipidaemia. The results demonstrated that the subjects who received 320 mg/day ACNs for 12 weeks showed further improvement in reducing serum IL-6 and tumour necrosis factor α (TNF-α), serum malonaldehyde (MDA), urine 8-iso-prostaglandin F2α (urine 8-iso-PGF2α), and 8-hydroxy-2′-deoxyguanosine (8-OHdG) than those who received 80 mg/day and 40 mg/day ACNs. On the other hand, Aboonabi et al. [102] sought to uncover whether ACN supplementation would ameliorate cardiometabolic abnormalities commonly associated with metabolic syndrome patients and inhibit the activation of platelet surface markers. The data obtained demonstrated that four weeks of anthocyanin supplementation significantly reduced cardiometabolic risk factors comprising the average serum fasting blood glucose (by 13.3%, *p* < 0.05) and lipid profiles by a significant reduction in triglyceride (by 24.9%, *p* < 0.05) and LDL- cholesterol (by 33.1%, *p* < 0.05) in the metabolic syndrome patients. Moreover, in females, the anthocyanin supplementation also decreased high sensitivity C-reactive protein level (by 28%, *p* < 0.05). Li et al. [99] demonstrated that cyanidin reduced lipopolysaccharide-induced mitochondrial reactive oxygen species (ROS) production. Moreover, cyanidin showed to be a more potent reductant of cytochrome C than ascorbate at the same concentration [98]. These authors proposed that cyanidin and specific flavonols combined with cytochrome C at low concentrations (few µM) were able to inhibit the pro-apoptotic cardiolipin-induced peroxidase activity of cytochrome C. In sum, the intake of berries as nutraceuticals or functional foods could be suggested for the prevention and control of CVDs.

### 2.2. Neurodegenerative Diseases

Neurodegenerative diseases including Parkinson’s disease, Alzheimer’s disease, and amyotrophic lateral sclerosis are symptomatically characterized by the impairment of cognitive or motor functions [111]. Dietary ACNs can enhance cognitive performance, exert neuroprotective effects against neurodegenerative disorders, and minimize cognitive decline, suggesting their potential application for the prevention of several neurological diseases (e.g., Alzheimer’s, Parkinson’s disease) (Table 3). Notwithstanding, the underlying mechanisms of their action are not completely recognized. The synaptic plasticity, neurogenesis, passage across the blood-brain barrier, modulation of cell signalling pathways and expression of genes involved in neuroinflammation, are some of the proposed mechanisms [112,113]. The ability of ACNs to change cell signalling pathways and expression of several different genes engaged in the regulation of numerous processes including inflammatory responses, redox balance, cell migration, and metabolism has been earlier demonstrated [113,114]. In this sense, Milenkovic, and collaborators [113] evaluated the effects of 12-week ACN-rich bilberry extract supplementation (0.02%) on global gene expression in the hippocampus of ApoE^−^/^−^ mice with the purpose of understanding the molecular mechanisms underlying anthocyanin neuroprotective effects. The results suggested that ACNs-rich bilberry extract altered the overall gene expression pattern in the hippocampus of ApoE^−^/^−^ mice. In addition, the bioinformatic analyses proposed that the identified 1698 differentially expressed genes could be involved in numerous cellular and molecular processes, comprising inflammation, neuronal function, metabolic processes, and signal transduction, which are involved in cognitive function and neurodegenerative disorders (e.g., Alzheimer’s, Parkinson’s disease). Furthermore, Vauzour et al. [115] elucidated the underlying mechanisms of actions associated with anthocyanin-rich extract, specifically neuronal signalling, and synaptic function/integrity. The results suggest that the consumption of anthocyanin-rich extract improves learning capabilities in aged rats, counteracting spatial memory loss in aged brains, through the modulation of several cell signalling events implicated predominantly in synaptic plasticity, apoptosis, and cytoskeleton remodelling. These behavioural variations are complemented by a regional rise of brain-derived neurotrophic factor mRNA expression within the hippocampus, highlighting a possible mechanism of action attributable to the memory enhancements detected in the aged animals [115].

ACN is well documented to have a protective effect on nerve tissue by crossing the blood-brain barrier. Amyloid-β toxicity and the modified amyloid precursor protein that process the potential aggregation of glycation products are key pathogenic features of Alzheimer’s disease [134]. Wen et al. [116] investigated the synergistic neuroprotective effect of metformin and cyanidin 3-*O*-galactoside (Cy3gal) by behavioural and histopathological assays and metabolite analysis in SAMP8 mice. The behaviour experiments showed that the SAMP8 mice treated with metformin and Cy3gal showed improved spatial learning and memory compared with the SAMP8 model group, which suggests their positive effect on postponing the progression of Alzheimer’s disease. Moreover, El-Shiekh et al. [126] demonstrated that *Hibiscus*—ACNs enriched extract prevents memory impairment, and this could be attributed to the amelioration of streptozotocin -induced neuroinflammation and amyloidogenesis. Moreover, according to these authors, *Hibiscus* represents a promising safe agent that can be repurposed for Alzheimer’s disease through exerting anti-inflammatory, anti-acetylcholinesterase, antioxidant, and anti-amyloidogenic activities. The effects of Cy3glu on M1/M2 polarization and the mechanism to regulate anti-inflammation and phagocytosis, both in vitro and in vivo, were investigated by Sanjay and collaborators [122]. The results demonstrate the effects of Cy3glu in shifting the M1/M2 polarization of microglia via activation of PPARγ and the TREM2-mediated enhancement of Aβ phagocytosis in Aβ42-treated HMC3 cells. Cy3glu not only has anti-inflammatory properties but also promotes eliminating accumulated β-amyloid by enhancing phagocytosis.

Parkinson’s disease is a result of the progressive loss of dopaminergic neurons in the substantia nigra [134]. Zaim et al. [119] investigated the potential neuroprotective effect of black carrots-ACNs enriched extract on human SH-SY5Y cells treated with 1-methyl-4-phenylpyridinium (MPP+) to induce Parkinson’s disease-associated cell death and cytotoxicity. The results suggest the first evidence that black carrots-ACNs enriched extract significantly protected SH-SY5Y cells from MPP+ induced neurotoxicity by inhibiting ROS mediated oxidative stress and apoptosis. Filaferro et al. [132] disclosed the antioxidant and neuroprotective activity of an ACNs-rich extract from Sweet Cherry (*Prunus avium* L.) using in vitro and in vivo models. The extract was demonstrated to be effective at a cellular level in reducing the cytotoxicity, intracellular reactive oxygen species (ROS) production in the cell lines tested (SH-SY5Y, BV2), after exposure to the neurotoxin *Drosophila melanogaster* rotenone. Moreover, the extract improved the resistance of the nematode *Caenorhabditis elegans* against thermally induced oxidative stress, mainly in young animals, suggesting it to be suitable for protection against oxidative stress.

Regarding to isolated ACNs, a study conducted by Chen and collaborators [125] and hippocampal metabolome alterations in aging rats demonstrated that petunidin-3,5-*O*-diglucosides (Pn3G5G) isolated from *Lycium ruthenicum* Murr. fruit enhanced biological pathways and processes, particularly biosynthesis of amino acids, ABC transporters, cysteine and methionine metabolism, protein digestion and absorption, biosynthesis of cofactors in the hippocampus of aging rats. In sum, Pn3G5G likely improves antioxidant and anti-inflammatory defences as well as keeps the normal function of the hippocampus by affecting these metabolic pathways, in that way alleviating cognitive impairment in aging rats. Furthermore, the disaggregation reaction of single amyloid β (Aβ) fibrils by delphinidin-3-galactoside (D3gal), cyanidine-3-galactoside (Cy3gal), and malvidin-3-galactoside (Mv3gal) was studied by total-internal-reflection-fluorescence microscopy with a quartz-crystal microbalance (TIRFM-QCM). The results demonstrated that D3gal promotes high disassembly ability; it completely dissolves Aβ amyloid fibrils, whereas Cy3gal interacts with Aβ amyloid fibrils, but it fails to show the disassembly activity on the fibrils. On the other hand, Mv3gal cannot even bind the fibrils. According to the authors, a possible explanation for these results is the number of hydroxyl groups in six-membered ring B, since Cy3gal and D3gal have two and three hydroxyl groups, and they interact with Aβ amyloid fibrils, whereas Mv3Ggal has only one hydroxyl group and fails to interact with the fibrils. Therefore, it was possible to conclude that a diet rich in ACNs can prevent neurodegenerative diseases.

### 2.3. Anticancer Effect

Oxidative damage to the cells and inflammation are two hallmarks triggering the development and progression of different forms of cancer. Therefore, the ingestion of compounds able to attenuate or disrupt these harmful processes may confer resilience to our organism against cancer if such molecules were able to reach the cells where the deleterious events are occurring. In this aspect, one of the protective roles of ACNs certainly lies in their antioxidant potential. For this reason, ACNs have been reported to exert therapeutic and preventive effects in almost every type of cancer, broadly by causing cytotoxic effects and inducing DNA damage and consequent cell cycle arrest [134,135]. We will, however, focus our discussion on the most recent reports in this field and the forms of cancer, with stronger evidence of protective effects elicited by ACNs, namely prostate cancer (PC), colon cancer (CC), namely lung cancer (LC), breast cancer (BC), and melanoma (Table 4).

Prostate malignancy is one of the most prevalent and lethal cancers affecting men worldwide. For this reason, the potential chemopreventive effect of ACNs in this form of cancer has been widely studied through in vitro approaches using different prostate cancer (PC) cell lines, as well as in vivo, using animal models, mostly rats and mice (Table 4). Regarding the in vitro evidence, extracts from different berries, such as *Vaccinium myrtillus* [136], *Lycium ruthenicum* Murray (black fruit wolfberry) [137], or sweet cherries [138], have been recently shown to increase the apoptotic rate and decrease cell viability of PC cell lines. But such an effect was also found using extracts from other foods, such as red cabbage juice [139] or the Ready to Serve (RTS) beverage prepared from *Ixora coccinea* fruits [140]. The cytotoxicity against human PC cells of the *Ixora coccinea* fruits is very relevant because this evergreen shrub is extensively used in Indian traditional medicine and so constitutes another cheap source of bioactive ACNs against PC in a huge population with limited access to proper health care services. Concerning the protective effects of ACNs against PC, involving in vivo models, recently Lamas et al. [141] observed a dose-dependent control of inflammation and oxidative-stress in the FVB mice fed with an extract of the Brazilian berry *Myrciaria jaboticaba*. A similar result was found by Kim et al. [142] that reported that the ACNs-rich extract of *Aronia melanocarpa* (black chokeberry) can attenuate the development of testosterone-induced prostatic hyperplasia in Wistar rats. Moreover, the authors have shown that such an effect was dependent on the abundance of the major ACNs present in the extract, Cy3glu and Cy3xyl Purple rice constitutes a promising functional food due to their rich bioactive composition, namely in ACNs. For this reason, the work of Yeewa et al. [143] is very relevant as the authors reported that the hexane insoluble fraction of this coloured rice retards carcinogenesis and castration-resistant cancer growth of the prostate and such effect occurred through the suppression of androgen receptor-mediated cell proliferation and metabolism.

Epidemiological evidence points to a protective effect of diets rich in fruits, vegetables, and whole grains against CC [144]. Therefore, ACNs, being abundant in most fruits and vegetables and responsible for their colour, will certainly contribute to mitigating CC development. This assumption has been largely supported by studies involving ACNs-rich extracts from fruits and vegetable foods as well as pure ACNs solutions (Table 4). Such studies were performed in CC cell lines, animal models, and clinical trials and evidence points that ACNs are largely responsible for such chemoprevention effects (reviewed in [145]). Moreover, such effects have been assayed in diverse types of CC cell lines, such as Caco-2, DLD1-1, HT-29, HCT-116, SW480, or SW620, therefore covering a wide range of types of cells associated with CC development and progression (Table 3). Beyond the protective effects reported against PC [142], ACNs from *Aronia melanocarpa* (black chokeberry) extracts were also reported to be effective against CC cells lines, inducing apoptosis in Caco-2 cells through the Wnt/beta-catenin signalling pathway [146]. A similar effect was observed by Gill et al. [147], which assayed three different species of chokeberries (purple, red and black, the last one being the *Aronia melanocarpa*). According to the data obtained, *Aronia melanocarpa* was the most effective against CC. Moreover, such enhanced effect in comparison with the purple and red chokeberry species assayed, correlated with the darker colour, higher overall phenolic content, antioxidant capacity, and presence of caffeic and chlorogenic acids in the black chokeberry. Regarding this observation, it is relevant to highlight the work from Mazewski et al. [148] that assayed eleven extracts from different fruits sharing unconventional and strong colours, namely red and purple grapes, purple sweet potato, purple carrot, black and purple beans, black lentil, black peanut, sorghum, black rice, and blue wheat. Overall, the authors report several protective effects against CC that include cell cycle arrest, apoptosis stimulation, and inhibition of anti-apoptotic proteins and tyrosine kinase. The attribution of these protective effects to the ACNs fractions of the different natural extracts is supported by the studies performed with pure ACNs such as Cy3glu and Dp, which evidence similar effects against CC development and progression [149,150,151,152]. The use of animal models provides additional evidence of the protective effects that ACN-rich extracts elicit against CC. In this regard, Fragoso et al. [153] have shown that the lyophilized açaí pulp (*Euterpe oleracea* Mart) attenuates colitis-associated colon carcinogenesis. Furthermore, the authors studied the main ACN from this fruit, cyanidin 3-rutinoside, and observed that it affects the motility of RKO CC cells [153]. Additional research recently reported involving CC rat models fed with ACNs-rich extracts points to important anti-inflammatory effects [154,155], providing additional evidence of the importance of inflammation in cancer development and progression.

One of the limitations pointed to the bioactive potential of ACNs is limited availability to protect cells as most polyphenols, including ACNs, are methylated in the gut and liver, therefore hindering their absorption. In this context, Grimes et al. [156] studied the effect of entacapone with ACNs. Entacapone is an inhibitor of the enzyme catechol-*O*-methyltransferase (COMT) which methylates polyphenols and so its use in combination with ACNs promoted a synergetic effect in the growth inhibition of CC and BC cells.

LC is one of the most prevalent and deadly worldwide and this certainly fosters Zhang et al. [157] to study the association between dietary ACNs and LC risk. Their epidemiologic study involving the dietary habits of about 10,000 Americans points to a positive correlation between dietary ACNs consumption and LC risk in this population. Different mechanisms should be involved in the protection offered by dietary ACNs against LC. Lu et al. [158], for instance, showed that the ACNs extracted from the fruits of *Vitis coignetiae* Pulliat, widely used in Korean folk medicine to treat inflammatory diseases and cancers, inhibit the expression of several transcription and growth factors that promote proliferation, angiogenesis, invasion, and migration of non-small cell LC (NSCLC) cells. Similar results were obtained when LC model cells were assayed with Cy3glu and Dp solutions, unveiling the downregulation of specific pathways [159] and transcription factors [160].

Melanoma is the deadliest form of skin cancer and its incidence significantly increased in the last decades. For this reason, research for chemopreventive agents against melanoma is very active and ACNs have great potential in this field. Wang et al. [161] recently studied the effect of ACNs-rich extracts obtained from blueberries on B16-F10 melanoma cells. The authors observed a dose-dependent inhibition of the B16-F10 cell’s viability and proliferation, as well as a cell cycle arrest at the G0/G1 phase and induced early apoptosis. Rugina et al. [162] also reported that the aqueous elderberry extracts exhibited a dose-dependent effect against melanoma cells by promoting cell integrity and apoptosis and inhibiting cell proliferation. The authors showed that these extracts are rich in several ACNs, namely C3-*O*-sambubioside, C3-*O*-sambubioside-5- glucoside, C3,5-digluc, cyhexoside-pentoside, and Cy3glu. In turn, Liu et al. [163] challenged mice and human melanoma cells with Cy3glu and reported this ACN inhibits tumorigenesis both in vitro and in vivo via the oestrogen receptor beta.

BC is certainly one of the forms of cancer that more impact has in our societies and so the obtention of a natural chemopreventive agent able to invert its incidence and mortality would be a remarkable achievement. Overall, BC treatment targets the inhibition of the mechanisms that regulate oestrogen activity. This can be attained either by antagonists of the oestrogen receptor (ER) or by the inhibition of oestrogen synthesis. In this context, preclinical studies in BC/CC rodent models point to a chemopreventive effect ACNs [164]. Recently, Han et al. [165] provided evidence that Dp suppresses BC by acting in the tumour suppressor microRNA-34a in cell lines and tumour tissues, but the results in human studies are contradictory. The EU-funded project ATHENA conducted during the last 10 years shows that dietary ACNs may protect against the damage caused by radiotherapy treatment for BC [166]. Following this result, Bracone et al. [167] studied if ACN supplementation could mitigate the skin toxicity caused by radiotherapy in BC patients. Upon the study involving 193 BC patients, the authors concluded that despite ACN supplementation being well tolerated and safe, it did not prevent RT-induced local skin toxicity. In another recent epidemiologic study involving over 1500 BC patients and 1500 controls in China, it was reported that intake of flavonoids, anthocyanidins, proanthocyanidins, flavanones, flavones, flavonols, and isoflavones was associated with a lower overall BC risk [168]. Overall, thi recent data about the role of ACNs in BC is not conclusive and further studies are required.

**Table 4:** The effects of ACNs in different forms of cancer.

Source/ACNs	Type of Study (Cell Line, Cancer Type/Mouse/Subjects)	Reported Effect	Ref.
Cancer type			
*Vaccinium myrtillus* berry extract	LNCaP (PC)	↑ apoptotic rate	[136]
*Lycium ruthenicum* Murray (ACN monomer, Pt3G)	DU145 (PC)	↓ cell proliferation, ↑ apoptosis, and cell cycle arrest in S phase	[137]
Sweet Cherry	PNT1A, LNCaP, PC3 (PC)	↓ cell viability	[138]
Red cabbage juice	LNCaP and DU145 (PC)	↓ cell viability	[139]
*Ixora coccinea* fruits	LNCaP, FGC (PC)	anticancer activity	[140]
Brazilian berry extract	Male FVB mice (PC)	dose-dependent control of inflammation and oxidative-stress in aging and high-fat diet-fed aging mice	[141]
*Aronia melanocarpa* containing Cy3glu and Cy3xyl	Wistar rats (PC)	Attenuated the development of testosterone-induced prostatic hyperplasia	[142]
ACNs-rich fraction from purple rice	Male heterozygous TRAP rats (PC)	Retarded carcinogenesis and castration-resistant PC growth (suppression of androgen receptor-mediated cell proliferation and metabolism)	[143]
Chokeberry extracts (ACNs)	Caco-2 cells (CC)	↓ cell growth; G1/G0 and G2/M phases arrest; ↑ p21WAF1 and p27KIP1 expression; ↓ cyclin A and cyclin B expression	[146]
Chokeberry extracts (ACNs and phenolics)	HT-29 (CC)	↓ cell proliferation	[147]
Coloured fruits and vegetables	HT-29, Caco-2, and HCT-116 cells (CC)	↓ anti-apoptotic proteins (survivin, cIAP-2, XIAP), ↑ apoptosis and G1 arrest, tyrosine kinase inhibition)	[148]
Cy3glu	HCT116, Caco2, and SW480 cells (CC)	Regulated the interaction of talin with β1A-integrin; promoted the attachment between CC cells and fibronectin; inhibited 3D spheroid growth	[149]
Dp	HCT-116 cell (CC)	↓ cell viability; promoted apoptosis; arrested G2/M phase; activated NFκB signalling	[150]
Dp	DLD-1, SW480, SW620 (CC)	inhibits human CC metastasis (↓ integrin and FAK signalling pathways, ↑ miR-204-3p upregulation)	[151]
Cy3glu and Dp3glu	HCT-29, HCT-116 (CC)	↓ PD-1, PD-L1	[152]
Lyophilized açai pulp	DMH and TNBS male Wistar rats (CC)	colitis-associated colon carcinogenesis attenuation; ↓ CC cells motility	[153]
Apple	azoxymethane CC rat model	Inhibited ACF, regulated apoptosis-related genes Aurka, p53 and COX-2, and cell migration-related genes MMP-2 and MMP-9	[169]
black soybean ACNs	Male Sprague- Dawley rats (CC)	Anti-inflammatory and antimicrobial effects, synergistic effect with ciprofloxacin in chronic bacterial prostatitis	[154]
Polymerized grape skin ACNs	Male Sprague- Dawley rats (CC)	Several protective effects against benign prostatic hyperplasia	[170]
Bilberry ACNs extract	AOM/DSS mouse (CC)	Prevents CC formation and growth	[171]
Lyophilized blackberries and strawberries	ACNs-rich sausages/AOM/DSS rat (CC)	↓ tumour number; ↓ pro-inflammatory gut bacterial	[155]
Strawberry and black raspberry extracts	AOM/DSS mouse (CC)	↓ tumour multiplicity; modulated the composition of gut commensal microbiota	[172]
*Prunus spinosa* drupes	HCT 116 cells, xenograft mouse (CC)	Inhibited growth and colony formation; promoted apoptosis in cells; ↓ tumour growth in xenograft mice	[173]
Table grapes with entacapone (Cy3glu, Dp3glu)	Caco-2, HT-29 (CC); MDA-MB-231 (BC)	↓ cell proliferation, ↑ extracellular ROS levels	[156]
Dietary ACNs	~10.000 participants (LC)	Positive correlation between dietary ACNs consumption and LC risk in Americans	[157]
*Vitis coignetiae* Pulliat ACNs	A549 (LC)	inhibit TNF-augmented LC proliferation, migration and invasion	[158]
Cy3glu	H1299 and A549 cells (LC)	suppresses LC progression by downregulating TP53I3 and inhibiting PI3K/AKT/mTOR pathway	[159]
Dp	A549 (LC)	inhibits angiogenesis through the suppression of HIF-1α and VEGF expression	[160]
Cy3glu	H661 (LC) xenografted into BALB/c nude mice	↓ large-cell LC growth & tumorigenesis	[174]
Blueberry fruits ACNs extracts	B16-F10 (melanoma cells)	dose-dependent inhibition of B16-F10 viability & proliferation, G0/G1 arrest, and induced early apoptosis	[161]
Aqueous elderberries extract rich in ACNs	250 µg/mL (melanoma cells)	↑ LDH; detachment, rounding up, shrinkage, and blebbing of membrane & apoptotic bodies;	[162]
Cy3glu	mice & human melanoma cells	↑ apoptosis, ↓ tumour growth & volume	[163]
Strawberry (Pg3glu)	N202/1A, N202/1E (murine BC cells)	↓ cell viability, ROS induction & mitochondrial damage	[175]
Dp	Female Sprague-Dawley (100 mg/kg rat/day (BC)	↑ tumour suppressor miR-34a in tumour tissues	[165]
1522 BC cases, 1547 control subjects (China)	Free choice meals (BC)	Inverse association between dietary ACNs ingestion and & BC risk	[168]
193 BC patients	water-soluble ACNs (125 mg) (BC)	ACNs supplementation did not prevent radiotherapy-induced local skin toxicity	[167]

Legend: ACNs—anthocyanins; BC—breast cancer; CC—colon (colorectal) cancers; Cy3glu—Cyanidin-3-glucoside; Cy3xyl—Cyanidin-3-xylose; DMH—1,2-dimethylhydrazine; Dp—delphinidin; Dp3glu—delphinidin-3-glucoside; LC—lung cancer; LDH—lactate dehydrogenase; Mv—malvidin; PC—prostate cancer; PD—Programmed cell death protein-1; PD-L1—programmed death-ligand 1; Pg3glu—Pelargonidin3-glucoside; TNBS—2,4,6-trinitrobenzene acid.

### 2.4. Diabetes Mellitus

The excessive accumulation of adipose tissue in the human body, known as obesity, is the strongest risk factor for developing diabetes, namely, type 2 diabetes mellitus (T2DM), which is responsible for 90% of all diabetic patients [176]. The term diabesity represents this crosstalk between obesity and diabetes sharing inflammatory processes as a hallmark. These deleterious processes can be prevented by natural antioxidants, such as ACNs. Previous epidemiological evidence points out that dietary ACN consumption correlates with the reduction of type 2 diabetes mellitus and improvement of glucose metabolism (reviewed in [177]). Moreover, such protection seems to be dose-dependent, demonstrating the protective potential of chronic dietary ACN intake [178]. Different mechanisms seem to be involved in such protection. In diabetes, there is an up-regulation of NFκB and ACNs inhibit this signalling pathway, leading to a reduction in oxidative stress and inflammation [179]. ACNs were also associated with the downregulation of the glucose transporters GLUT2 in human intestinal Caco-2 cells [180,181]. This result is certainly related to the observation of diminished intestinal glucose absorption in patients and better postprandial glycemia in healthy populations [182,183]. In turn, Tani et al. [184] treated diabetic human aortic endothelial cells with Dp-3-rutinoside-rich blackcurrant extract and observed an increase in the secretion of glucagon-like peptide-1 which improved glucose tolerance. Other works involving mouse models provide evidence that the consumption of ACNs-rich extracts, such as those obtained from raspberry [185] and black currant [179], prevents diet-induced obesity by mitigating oxidative stress and modulating hepatic lipid metabolism. Also using high-fat diet-induced obese mice, Wu et al. [186] reported that ACNs in black rice, soybean, and purple corn increase faecal butyric acid, an anti-inflammatory molecule with regenerative properties in the intestines, and prevent liver inflammation. In a recent pilot study, Azzini et al. [187] recruited 11 overweight or obese women which received 500 mL/day of a commercial red orange juice rich in ACNs for 12 weeks. The authors report that the intervention resulted in a decrease in total cholesterol and LDL cholesterol, but no significant effects on obesity, insulin resistance, or inflammatory status. Further studies are therefore necessary to clarify the role of dietary ACNs in the prevention and management of diabetes.

### 2.5. Visual Health

The awareness of the protective effects ACNs elicit on visual health is not new. During World War II, for instance, bilberry jam was administered to British pilots to improve their night sight [57]. In early 2000, Kajimoto et al. [188] performed an interventional study with primary school students with pseudomyopia and showed that ACNs from bilberry (*Vaccinium myrtillus* L.) promoted recovery of visual acuity. This and many other protective effects attributed to ACNs for vision and eye health are eventually related to their ability to absorb light in the UV (280–400 nm) and blue light region (360–500 nm). Effectively, ACNs are the only class of polyphenols able to do that, which could protect human retinal cells against light-induced damage (reviewed in [189,190]) or more broadly against the excessive production of ROS and consequent oxidative damage to surrounding cells. Evidence points to crosstalk between this oxidative damage and inflammation which in turn affects the integrity of the blood-retinal barrier (BRB) and induce pathological neovascularization [189,191]. Overall, oxidative damage to the cells is a hallmark triggering the onset and progression of different ophthalmological diseases, such as glaucoma, age-related macular degeneration, and different retinal degenerative diseases (RTDs) [189,190,192]. Glaucoma, for instance, is one of the leading causes of irreversible blindness, often triggered by an increase in intraocular pressure. This eventually damages the optic nerve and retinal ganglion cells, ultimately leading to visual field dysfunction. While several phenolics, such as baicalein, forskolin, marijuana, ginsenoside, resveratrol, and hesperidin, were shown to reduce intraocular pressure, ACNs elicit neuroprotective effects on retinal ganglion cells. Such an effect seems to involve different mechanisms, especially antioxidant, anti-inflammatory, and anti-apoptosis mechanisms (reviewed in [190,193]). Age-related macular degeneration (AMD) is another major cause of blindness. It is the most prevalent cause of the condition in elderly populations, and there is no effective treatment for its dry form. For this reason, the protective effect of the fruit extract of *Aronia melanocarpa* on rat retina is so relevant. Using a NaIO3-induced dry AMD model, Xing et al. [194] showed that these ACNs-rich extracts protect the rat retina, mitigating the effect of oxidative damage and upregulating at least five crystallin proteins which protect the retina ganglion cells.

To elicit the protective effects, ACN would need to pass thorough the blood-aqueous barrier and BRB, and this ability has been shown in rats and rabbits [195]. Moreover, ACNs were reported to accumulate in ocular tissues as their total concentration in these tissues was found to be higher than that measured in plasma upon ACNs intravenous or intraperitoneal administration [193]. The route to explain this result seems to involve facilitated transport via glucose transporters, namely the constitutive isoform GLUT1 [196].

The protective effects observed in different studies using ACNs-rich extracts prompted research using purified ACNs to try to ascribe to ACNs the reported effects. Cy3glu as one of the most abundant ACNs was shown to mitigate the photooxidation-induced apoptosis and angiogenesis in the retina [197], possibly through the activation of Nrf2/HO-1 pathway and NFκB suppression [198]. In turn, the same suppression of NFκB activation together with cox-2 expression seems to be the mechanism involved in the protection of the lens epithelial cells to cope with apoptosis induced by high glucose levels, therefore preventing the development of cataracts [199]. Similarly, another important ACN, malvidin promotes SOD and catalase expression in high glucose-induced human retinal capillary endothelial cells. Such activation protects these cells not only from oxidative stress-induced damage but also involves anti-inflammatory mechanisms through the inhibition of ICAM-1 and NFκB [200]. These selected examples are clear evidence of the protective effects ACNs have on our visual health and for this reason, a growing number of applications and products are incorporating these bioactive compounds in health care products targeting our vision.

## 3. Potential Applications

**Figure 4:**
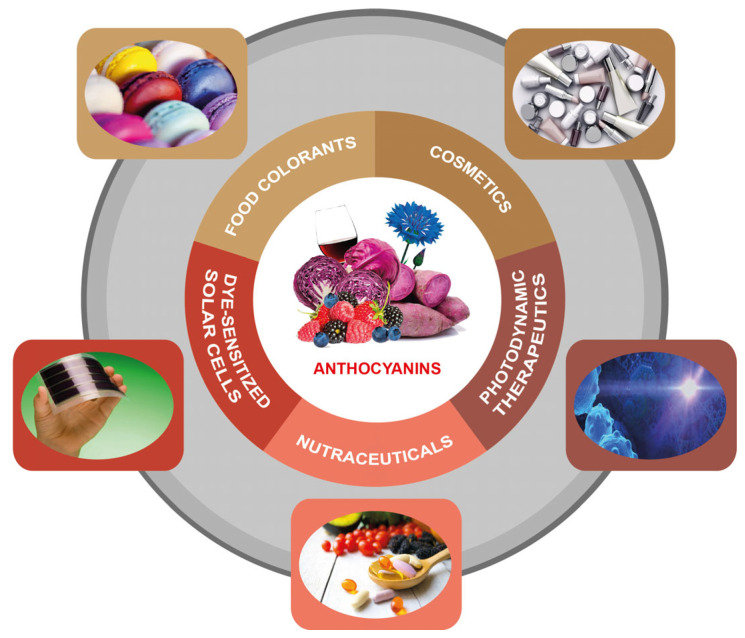
Biomedical and technological applications of anthocyanins.

ACNs have a huge range of biomedical applications in functional foods, cosmeceuticals, and pharmaceutical products, as well as applications in a more technological field due to their photochromatic properties being used in replacement of synthetic dyes (Figure 4).

### 3.1. Food Colorants

ACNs have been long implied as suitable food colourants due to their photochromic characteristics. Consequently, these natural pigments have been deeply studied for their characteristics in such areas over the years.

In a more recent period, the most relevant focus has been on how to improve their inner characteristics to originate colours with higher stability and products with a higher shelf-life time. Therefore, herein we present the most recent studies regarding different approaches to enhancing ACNs properties for food-colourant areas.

One of the main issues about ACNs’ colour is the dependence on pH. The colour of these compounds can range from bright red to purple and bright blue depending on the pH environment. However, at basic pH’s, where the purple and blue colours are achieved due to the predominance of neutral and anionic quinoidal structures, the stability of the ACNs is highly decreased. Also, in the context of the food industry and nutrition, most products have a range of pH values from moderately acid to moderately basic. Therefore, in this range, the colour of ACNs tends to fade due to the hydration of the core structure and the formation of the hemiketal form [201]. On the other hand, ACNs are also prone to degradation by exposure to high temperatures, light, oxygen, enzymes, proteins, or metal ions [15]. One of the major approaches to prevent the loss of structure and colour, mainly by the control of kinetic and pH parameters, has been the encapsulation of ACNs using different techniques. Very recently, ACNs from red cabbage were used to produce microparticles of maltodextrin and Arabic gum rich in these compounds using spray drying [202]. The authors found that the ideal ratio for encapsulation was a ratio of 25:25 of maltodextrin:arabic gum. They verified that the temperature resistance of the microparticles produced was higher than the normal storage temperature (up to 40 °C), concluding that these formulations may be suitable for industrial applications.

In another study, the impact of grape pectic polysaccharides on malvidin-3-*O*-glucoside thermostability was assessed. The authors verified that the thermal stability of these ACNs was greatly increased upon the formation of the encapsulated particles. Moreover, the results also showed that less branched structures and pectin flexibility are key factors in the promotion of the stability of Mv3G [203]. These results suggest another potential system for use in the food colourants industry for the stabilization of ACNs.

The possibilities of encapsulating systems are immense, and many different combinations can be found in the literature. Such a fact prompts the high potential of such an approach for the food industry.

A very recent study has used a *Melodorum fruticosum* Lour ACN-rich extract to be incorporated in maltodextrin-Arabic gum particles using a double drum technique [204]. After the successful production of the nanoparticles, the authors incorporated them in a food model (gummy jellies) and verified that the shelf-life time improved considerably, up to 8 weeks.

On the other hand, nanoliposomes have been recently used to encapsulate ACNs from red cabbage. This procedure showed to greatly improve the storage stability of the ACNs and improve their release on lipophilic food models, revealing an interesting approach for the applications of these hydro soluble compounds in non-water-soluble matrices [205].

Co-pigmentation with different molecules has also shown to be an interesting strategy to prevent the degradation of ACNs and the maintenance of their inner characteristics. The addition of other phenolics, polysaccharides, or proteins showed to improve the stability of ACNs [206]. These copigmented complexes usually adopt a configuration of vertical stacking, preventing the formation of the hemiketal structures (colourless form) [207].

Recently, different studies tested the copigmentation effect of different molecules on ACNs stability, including organic acids, flavonols, and amino acids [208,209,210]. In all the studies, a protective effect resulting from the copigmentation was verified, however different ratios and efficacies were also observed, suggesting that not only the type of copigment but also the type of ACNs are important for determining the success of the copigmentation processes. For instance, in the case of the organic acids, the acylation of the ACNs reduced the copigmentation rate due to steric hindrance while in the case of the amino acids (aspartic acid) the number of sugars attached to the ACNs was proportional to the decrease in the copigmentation efficacy [208,210].

Other molecules such as fatty acids have also been shown to be able to form copigmentation complexes with ACNs from purple sweet potatoes [211]. Interestingly, ACNs from purple sweet potatoes are highly acylated and glycosylated [212]. Therefore, the complex formation seems to highly depend on both sources.

Besides the techniques utilized to increase the stability of ACNs, the use of complex ACNs can also represent a wise strategy for the food industry. These ACNs, from sources such as purple sweet potato, red cabbage, or butterfly pea flowers, are highly glycosylated and acylated, conferring enhanced stability [201,213,214,215].

### 3.2. Dye-Sensitized Solar Cells

In the last few years, alternative energy sources to fossil fuels have been one of the most studied fields in scientific research. In this regard, researchers embarked on the design of new technologies able to harvest energy, where solar cells were pivotal for the success of this area [216,217]. Among solar cells, a particular kind has received growing attention, due to its cost-effectiveness and resource-unlimited characteristics: dye-sensitized solar cells (DSSCs). These systems consist of a photoanode, a counter electrode, and an electrolyte, being able to convert radiation into electricity by sensitization wide band gap semiconductors with dyes as photosensitizers [218].

These semiconductors are usually metal oxides with excellent photochemical and optoelectronic characteristics such as cadmium oxide (CdO), titanium dioxide (TiO_2_), and zinc oxide (ZnO), among others. However, most of the successfully produced DSSCs, utilize synthetic dyes, mainly derived from ruthenium metals due to their intense charge-transfer absorption in the visible range of the solar spectrum, long excitation lifetime, and highly efficient metal-to-ligand charge transfer [219]. The problem lies in the several disadvantages that shave these compounds, namely, related cost, resource scarcity, heavy metal toxicity, complex synthesis, and low yield.

To overcome these issues, alternatives to synthetic options have been in the scope of researchers over the years. In this context, ACNs were revealed to be interesting candidates as multistate systems for information processing at the molecular level, due to their physical, chemical, and structural characteristics.

Herein, we sum up the latest developments in the use of ACNs from different sources in this technology field.

One of the flagships of ACNs is their structural pH dependence. It represents their two-sided coin characteristics, where this can be extremely advantageous in some cases, while in others, it is a major flaw. Bearing this in mind, very recently, Mejica and colleagues, have investigated the effects of pH on the stability of ACNs as a photosensitizer for DSSCs [219]. They utilize ACNs from Malabar spinach ripped fruits and tested the impact of different pH values (1, 2, 3, 4, 5, 6, 7, 8, 9, 11, 11.33) on their electrical properties. The results showed that in acidic conditions (from pH 1–6), the extract reached its maximum power efficiency at pH 2 (0.0029 mW/cm^2^). However, in acidic conditions, TiO_2_ particles that compose the DSSCs may be damaged overtime, therefore, the efficiency in basic conditions (pH 7–11.33) was tested. The authors found that the ACNs of Malabar spinach had an overall higher performance at alkaline conditions, with pH 9 as the ideal condition, with a produced power density of 0.0036 mW/cm^2^. At this pH, ACNs are mainly in their anionic quinoidal structure, suggesting the negative charges may promote energy conduction.

In other recent studies, the authors utilized ACNs from the Cassava plant (mainly composed of Cyanidin and Delphidins-3,5-*O*-diglucosides) and verified that the best performance was achieved at pH 8 [220]. However, a constant decrease was noticed from pH 2 to pH 6. Also, at pH 9 to 12, the efficiency was not as high as at pH 8. This suggests that the flavylium cations and anionic quinoidal bases have an important role in the efficiency at DSSC’s level.

Indeed, other studies reported that ACNs from other sources, namely *Syzygium cumini* and red cabbage, had their highest performance at low and around 8 pH levels [221,222,223].

Another important factor that may modulate the efficiency of ACNs at this level is their concentration. In a recent study, the influence of such a factor on electron transport in DSSCs was evaluated with ACNs from *Acanthus pubscenes* flowers (mainly composed of Cy3glu) [224]. The concentrations of ACNs utilized were 0.4, 0.6, 1, and 1.18 mg/mL, with solar cells conversion efficiencies of 0.065%, 0.111%, 0.140%, and 0.145%, respectively. It was observed that different factors were also improved with the increase in ACNs concentration such as open circuit current density, open circuit voltage, fill factor, and solar conversion. The authors attributed these phenomena to higher absorption of the light by the ACNs at higher concentrations, with more electrons being excited and injected into the conduction band of TiO_2_.

Other recent studies, including comparisons with other pigments, had also attributed interesting features to the ACNs [225,226,227]. Nevertheless, the use of ACNs focused on this area is still very recent with a great opportunity for growth and development.

### 3.3. Nutraceuticals

The perception of how fundamental health is and how problematic and consequent the disease state is increasingly evident in today’s society. The perception of human vulnerability to diseases, whether communicable or non-communicable, is increasingly a priority. The pandemic that surprised us in 2019 affected us in such a global way, it shook the conscience and security of the human being, reaching the point of the survival of the healthiest. If there is an increasing awareness of humans for the need to maintain health, and physical and mental care, there is also an increasing expansion of the availability and diversity of dietary supplements available to the consumer. In this topic, a particular focus will be given to supplements with preventive/pharmacological proven activity.

Nutraceuticals are nutrients that can modulate physiological and pathophysiological molecular mechanisms, thus having favourable health outcomes. These results can be achieved by pharmaceutical supplements or by the incorporation of extracts rich in bioactives in processed foods.

In the case of ACNs, there are several clinical trials related to cardiovascular health and cognitive performance of these bioactives, although few use commercial trade products.

The berry extract, which is marketed as a dietary supplement under the brand name Medox^®^, has 1 capsule containing 80 mg of Scandinavian bilberries and black currants ACNs. Medox^®^ is clinically proven through scientific studies at universities and university hospitals nationally and internationally since the product came on the market in 2000. These studies have been published in different highly respected scientific, internationally recognized nutritional- and medical journals [228,229].

Another example is standardized bilberry extract (*Vaccinium myrtillus* L.), with the trade name Mirtoselect^®^ and is characterized by a very specific and consistent HPLC profile that represents the “fingerprint” of the extract. The extract contains more than 36% of ACNs plus the full range of the non-ACNs components critical for its efficacy. Moreover, bilberries cannot be cultivated and are therefore picked from wild plants growing on publicly accessible lands exclusively between July and September. To be used as a nutraceutical, its necessary to ensure the quality, traceability, and authenticity of the extract. That is achieved by DNA analysis during quality control procedures, taking the fingerprint of the berries. This small edible berry whose use in traditional medicine has been documented since the Middle Ages became one of the major herbal remedies after the 16th century. Later studies have then identified some of the bilberry biologically active substances, particularly the ACNs, with impressive antioxidant properties, one of the proposed mechanisms of action [230]. Supported by epidemiological surveys, animal models and pilot clinical studies suggest this extract contributes to vascular and eye health efficacy, memory optimization, cardiovascular health, metabolic health, and more, with a recommended dose of 160–320 mg/day [231,232,233].

The exceptional features of Myrtoselect^®^ have attracted pharmaceuticals to create derived extracts with additional applications. Mirtogenol^®^ is a combination of Mirtoselect and French maritime pine bark extract which is called Pycnogenol. In a clinical trial with 20 asymptomatic subjects with intraocular hypertension, Mirtogenol^®^ was consumed in the form of tablets (80 mg of Mirtoselect^®^ and 40 mg of Pycnogenol), twice a day for 6 months. At the end of the study, the intraocular pressure decreased from 26 mmHG to 22 mmHG [234].

The European Commission’s Concerted Action on Functional Food Science, coordinated by the International Life Science Institute, has defined “functional food”. It is “a food that, together with the basic nutritional impact, has beneficial effects on one or more functions of the human organism thus either improving the general and physical conditions or/and decreasing the risk of the evolution of diseases” [235]. The amount and form of intake of the functional food should be as normally expected for dietary purposes.

Although there are commercially available examples of ACNs extracts that follow these criteria, there are no authorized health claims from the US Food and Drug Administration (FDA) or the European Food Safety Authority (EFSA) referring to ACNs.

Generally, the health benefits of ACNs supplementation to the consumer result from the interpretation of the numerous studies found in the literature investigating the bioactivity of ACNs both in vitro and in vivo. This method should be taken with special care since the upper-described standardization in Medox^®^ or Myrtoselect^®^ is normally not applied.

Besides pharmaceutical supplements, ACNs can also be incorporated into different types of foods, including bread, cookies, and pasta, by adding fruit or vegetables coloured extracts to the recipe or by using pigmented grains, transforming them into functional foods [236]. For example, red rice is used as a functional food to treat abdominal pain and cardiovascular disease [237,238]. Purple Canadian wheat has been used by Gardenia company to produce purple bread containing 13 major ACNs and cyanidin 3-glucoside as the predominant one [239]. This purple wheat is also used for noodles pasta fabrication and made commercially available by Singapore company Koka^®^. Some in vivo studies already suggest that the incorporation of coloured wheat (especially black wheat) in the diet can prevent obesity and related metabolic complications [240]. Besides, coloured wheat, which is biofortified with zinc, can fight malnutrition among children, one of the big health challenges in developing countries [241]. In addition, ACNs biofortified black, blue, and purple wheat exhibited lower amino acid cooking losses than white ones [242].

### 3.4. Cosmetics

Cosmetics products include deodorants, hair dyes, hair styling products, make-up, sunscreens, nail colourants, skin & hair care products, and skin & hair cleansing products, amongst others. Interest in natural ingredients is significantly increasing among cosmetic consumers in general. In response to these consumer interests and driven by sustainability issues, brands are introducing their lines organically cultivated extracts, with ACNs normally being associated with products that promise a healthy ageing outcome.

On this topic, the concern about the toxicological effects of synthetic dyes. As an alternative approach, renewable waste blackcurrant (*Ribes nigrum* L.) fruit skins from the fruit pressing industry can be extracted using acidified water. Intense, blue-coloured dyeing on hair could be achieved with λ_max_-vis at 580 nm, typical of the anionic quinonoid base. Hair provides an environment that enables the stabilization of the anionic quinonoid base on adsorption through association with cations in the hair and copigmentation effects [243].

Skin care formulations with antioxidants are being widely explored for their benefits to human skin. Even though ACNs appear to be appropriate ingredients for this type of application, undesirable colour changes might occur under pH variations. These water-soluble natural pigments are very unstable with changes in pH, temperature, and light, limiting their usage. One strategy that may be used to promote their use is the combination with clay minerals, broadening their application as biocosmetics [244].

Another approach is the inclusion of ACNs in emulsion systems. Recently, stable water in oil (*w*/*o*) emulsion containing 3% ACN extract derived from the peel of *Malus dosmestica* fruit was prepared, which kept the stability profile at different storage conditions over 90 days period [245]. Besides, ACN-containing formulations have been found efficacious (*p* < 0.05) for their effects on melanin content and skin erythema [245].

The application of ACNs as sunscreen filters was explored with purple sweet potato TNG73 extracts. At a concentration of 0.61 mg/100 g cream, ACNs absorbed 46% of all incident UV radiation; therefore, these compounds might be effective solar protectors to prevent skin damage [246].

This effect was also recently explored using cyanidin- and malvidin-3-*O*-glucosides pure standards and also elderberry and red wine ACNs extracts [247]. Overall, most of the compounds were found to exhibit UV-filter capacity, attenuate the production of reactive oxygen species in human skin keratinocytes and fibroblasts and showed inhibitory activity of skin-degrading enzymes (Tyrosinase, elastase, hyaluronidase, and collagenase) in the absence of cytotoxic effects [247,248]. Considering the described features of these compounds, a formulation containing them would benefit from a dual function of photoprotection blended with an anti-aging effect. In a more preventive way, the application of ACNs from red grapes and chokeberries to melanoma cells reduced proliferation, and diminished mitochondrial membrane potential and oxidative damage, all with no adverse effects on normal skin cells [249].

Also, keratinocytes pre-treated with ACNs extracts from red wine and blackberry during 24 h were able to better recover from wound induced by high frequency energy, reducing the recovering time by more than half [250].

Based on the literature evidence and on their research and development unit’s results, several cosmetic brands have products that include ACN extracts, especially highlighting their antioxidant properties.

Nivea^®^ brand is a great example of this, launching several cosmetic products such as face scrubs, shower gels and lip butter, which contain in their ingredients list *Vaccinium myrtillus* fruit extract and *Rubus idaeus* juice. Also, the Oriflame Swedish brand wants to increase the level of naturality of their products and is working in a Love Nature range with soaps, conditioners, body creams and body lotions that include different berries extracts in their formulations. In the L’Oreal group, Vichy Idéalia’s antioxidant complex is powered by blueberry extract.

### 3.5. Photodynamic Therapeutics

Photodynamic therapy has emerged in the last few years as a consolidated technology for the treatment of a wide range of pathologies.

The principle behind PDT is based on the activation of certain compounds (photosensitizers) by light after their local or systemic application and accumulation. The process is based on photoactivation through the absorption of light at suitable wavelengths, and the triggering of biochemical processes that culminate in the destruction of the damaged tissue. This phenomenon occurs by two distinct mechanisms that mainly depend on the intracellular quantity of oxygen. The photosensitizer is irradiated, after entering the cells, with the corresponding light absorption wavelength, which allows its excitation. The energy is then directed through the formation of a quantum of fluorescence while the remaining directs the photosensitizer to the triplet state, which represents the bioactive and therapeutic form of the molecule [251].

In type I, direct-contact reactions occur by the transfer of electrons or hydrogen between the involved actors (PS and the tissue) leading to the initiation of radical chain reactions and direct damage to the biomolecules. This happens due to the photosensitizer’s triplet state that can transfer energy to the biomolecules.

This subatomic particle transfer will create free radicals, and both the PS and substrate anions can react with the oxygen molecules present in the damaged tissue, creating ROS and culminating in oxidative stress and the destruction of the damaged cells.

The type II mechanism involves the transfer of the excitation energy directly to the triplet oxygen molecules originating singlet oxygen (^1^O_2_) species. ^1^O_2_ is highly reactive due to its electrophilic behaviour being capable of damaging different cellular components such as proteins, membranes, or even genetic material [189,252]. Both mechanisms result in the production of ROS, and due to these species’ short lifetime, PDT can produce a strong localized effect [189]. The research for PS alternatives to the ones existing so far, such as protoporphyrins and other molecules with a tetrapyrrole structure in their core, has led to an increased interest in ACNs and their derivatives. To meet the conditions for application on PDT, a molecule must absorb at wavelengths near the red light (600–800 nm) and be excited. In fact, ACN at the physiological pH exhibit quinoidal species, which absorb towards the red region of the light spectrum [201,253]. Furthermore, these compounds have also been shown to be excited and to produce charge-transfer reactions, as in the case of DSSC, previously discussed.

In a recent study, Cy3glu was utilized to mediate a photodynamic therapy against biofilms of *Porphyromonas gingivalis* [254]. The results that green laser light was able to induce the reduction in CFUs counting, upon the application of the ACN. However, this result was not as effective as in the case of the other tested compound, erythrosine. The authors then tested a concomitant application of both compounds and verified a synergistic effect suggesting that Cy3glu may have an interesting role in bacterial PDT.

In another study, photodynamic therapy mediated by açai oil (*Euterpe oleracea* Martius) was assessed for the potential treatment of melanoma, using a LED light source with a wavelength of 660 nm [255]. *Euterpe oleracea* Martius is rich in Mv and cyanidin derivatives [256], and the results showed that the application of the oil followed by photosensitization successfully killed 85% of melanoma cells. Also, in PDT-treated mice, an 82% tumour reduction was observed in comparison to the control group.

Understanding the specific action mechanisms of the photosensitizers is crucial for their potential applications. In that perspective, *Syzygium cumini* ethanolic extracts (rich in 3,5-*O*-diglucosides ACNs) were evaluated for their capacity to produce ROS from photogeneration under visible light radiation. The results showed that the extract was able to produce ^1^O_2_ under illumination with a light source [257].

Interestingly, ACNs are reported as oxygen singlet quenchers. Cy3glu, C3R, Cy3glual, Mv3G, and malvidin-3,5-*O*-diglucoside showed the capacity to function as quenchers of ^1^O_2_ [258]. This fact may set ACNs towards type I PDT, where their triplet state can react directly with the biomolecules [189].

Only a few approaches have been developed during the last few years regarding ACNs and PDT; however, these first results point to ACNs as an interesting target for use as new photosensitizers.

## 4. Concluding Remarks

In summary, ACNs constitute a large group of phytochemicals which are present in various fruits (such as red wine and red and black grapes, pomegranates, and plums) vegetables, coloured grains, and other foods. Beyond its potential applications in several technological and industrial fields, its use as natural dyes in the food and textile industry, being an excellent alternative to the conventional colourants, as well as a photosensitizer for dye-sensitized solar cells (DSSCs), nutraceuticals, cosmetics, and photodynamic therapeutics (PDT), they exhibit strong biological activity that can be used for preventing several diseases, including some metabolic diseases, cancers, CVDs, diabetes, and microbial infection, as demonstrated in several in vitro and in vivo studies, reported in the review. The typical mechanisms of action of these biologically active water-soluble phenolic pigments in prevention of diseases includes COX and MAPKs pathways, inflammatory cytokines signalling, free-radical scavenging, as well as changes in blood biomarkers. However, future research activities that involve human trials are required to ensure a greater impact of ACNs as acceptable therapeutic agents.

## Figures and Tables

**Table 1:** Concentration of ACNs in fruits and vegetables (fresh weight; FW).

Source	Max ACN Amount (mg Cy3glu/100 g)	Main ACNs	Ref.
Fruits			
Açaí	295	Cy 3,5-hexose pentose; Cy3glu; Cy3rut; Pg3glu; Pn3glu; Pn3rut; Cy 3-(acetyl)hexose	[66]
Apple	71	Cy3gal; Cy3glu; Cy3arab; Pn3gal; Cy7arab; Cy3xyl	[62,67]
Blackberries	200	Cy3glu; Cy3rut; Cy3xyl; Cy 3-malonylglucoside; Cy 3-dioxalylglucoside;	[61]
Black currants	219	Dp3glu; Dp3rut; Cy3glu; Cy3rut	[61]
Blueberries	407	Dp3gal; Dp3glu; Cy3gal; Dp3arab; Cy3glu; Pt3gal; Cy3arab; Pt3glu; Pn3arab; Mv3gal; Mv3glu	[68]
Chokeberries	356	Cy3gal; Cy3arab; Cy3glu; Cy3xyl	[69,70]
Cranberries	207	Cy3gal; Cy3glu; Cy3arab; Pn3gal; Pn3glu; Pn3arab	[71]
Elderberries	317	Cy3glu; Cy 3-*O*-sambubioside	[69,72]
Fig	11	Cy3glu; Cy3rut; Cy 3-sambubioside-5glu; Cy 3,5diglu	[73,74]
Grapes	186	Cy; Dp; Mv; Pn; Pt3glu; Mv; Pn; Pt 3-*O*-coumarylglu	[75,76]
Plums	37	Cy3xyl; Cy3glu; Cy3rut; Pn3rut; Pn3glu; Cy3gal; Cy 3-(6”-acetoyl)glu	[62,77]
Pomegranate	2000	Dp 3,5diglu; Cy3glu; Cy 3,5diglu; Pg3glu; Pg 3,5diglu	[78,79]
Peaches	2.5	Cy3rut and Cy3glu	[80,81]
Red cabbages	185	Cy 3diglu-5glu; Cy 3sindiglu-5glu; Cy 3-(p-coumaroyl)-diglu-5glu	[82,83]
Red raspberries	68	Cy 3-*O*-sophoroside; Cy 3-*O*-(2″-*O*-glu)rut; Cy3glu, Cy3rut; Cy 3-*O*-(2″-*O*-xyl)rut; Pg 3-*O*-sophoroside; Pg3glu; Cy 3,5-*O*-diglu	[69,84]
Strawberries	60	Pg3glu; Cy3glu; Cy3rut; Pg3rut; Pg 3-(malonoyl)glu; Pg 3-(6″-acetoyl)glu; Cy 3-sophoroside	[62]
Sweet cherries	463	Cy; Dp; Pg; Pn 3-*O*-rutinosides and glucosides; Cy 3-coumaroyl-diglu; Cy 3-*O*-sambubioside; Cy 3-5diglu; Cy 3-sophoroside; Cy3arab; Mv 3-*O*-glu-acetaldehyde	[85]
Tart cherries	82	Cy; Cy 3-*O*-sophoroside; Cy 3-glurut; Cy3glu; Cy3rut; Pn3rut	[69,86]
Tomato	7	Dp glu; Dp rut; Dp p-coumaroyl-rut; Dp p-coumaroyl-rut-glu; Mv 3-glu; Mv rut; Mv p-coumaroyl-rut-glu; Pt rut; Pt p-coumaroyl-rut; Pt p-coumaroyl-rut-glu	[87]
Vegetables			
Black carrot	23	Cy 3-(p-coumaroyl)-diglu-5glu; Cy xylglcgal; Cy cafxylglcgal; Cy xylgal; Cy phbxylglcgal; Cy sinxylglcgal; Cy ferxylglcgal;	[88]
Eggplant	6	Dp 3-(p-coumaroylrut)-5glu; Dp3glu; Dp 3glu-rhamnoside; Pt3rut; Cy3rut	[63,65]
Purple sweet potato	42	Pn 3-*O*-sophoroside-5-*O*-glu; Pn3glu; Cy 3-phb-sophoroside-5glu; Pn 3-phb-sophoroside-5glu; Cy 3caf-sophoroside-5glu; Pn 3caf-sophoroside-5glu; Cy 3caf-phb-sophoroside-5glu; Pn 3-dicaf-sophoroside-5glu; Pn 3caf-phb-sophoroside-5glu; Pn 3caf-fer--sophoroside-5glu;	[61]
Red Chicory	39	Cy3glu; Cy 3-*O*-(6″-malonyl-glu)	[61]
Red onion	30	Cy3glu; Cy 3-*O*-laminaribioside; Cy 3-(6″-malonyl-glu); Cy 3-(6″-malonyl- laminaribioside); Cy 3-xyl-glu--gal; Dp 3,5diglu	[68]

Legend: arab—arabinose; caf—caffeoyl; Cy—cyanidin; Dp—delphinidin; fer—feruloyl; glu—glucoside; gal—galactoside; Mv—malvidin; Pg—pelargonidin; phb—*p*-hydroxybenzoyl; Pn—peonidin; Pt—petunidin; rut—rutinose; sin—sinapoyl; xyl—xylosyl.

**Table 2:** The effects of anthocyanins in CVDs.

Source/ACNs	Study Type	Effects and Potential Mechanism	Ref.
Berries/ACNs supplement	In vivo, ACNs 40 mg/day, 80 mg/day, or 320 mg/day for 12 weeks	A positive correlation was observed between the changes in the urine 8-iso prostaglandin F-2α, 8-hydroxy-2′-deoxyguanosine levels and serum interleukin-6 levels in subjects from ACNs groups after 12 weeks of treatment	[101]
Berries/ACNs supplement	In vivo, 80 mg of ACNs on 2 different occasions for 28 days	Significantly decrease the cardiometabolic and adenosine diphosphate-induced platelet activation	[102]
Blackcurrant/ACNs-rich extract and isolated ACNs	In vitro, cells were treated with 0.5 or 1.0 µL of blackcurrant extract and 10 µM of ACNs for 3 months	The endothelial NO synthase mRNA expression and NO synthesis in human endothelial cells significantly increase	[100]
Blackcurrant/ACNs-rich extract	In vivo, the participants were fed 1.87 mg of ACNs/kg bodyweight for 1 week	Acute ingestion of a single dose of blackcurrant extract-maintained forearm blood flow, as well as forearm vascular resistance during an extended period of sitting	[103]
Blackcurrant/ACNs-rich extract	In vivo, the participant ingested 600 mg of blackcurrant extract/per day for 1 week	Decrease in systolic and diastolic blood pressure	[104]
Black Raspberry/ACNs-rich extract	In vivo, the rats were fed with 0.6% of black raspberry extract for 8 weeks	Intake of black raspberry extract alleviates hypercholesterolemia and hepatic inflammation induced by excessive choline with a high-fat diet	[105]
Blueberry/ACNs-rich extract	In vivo, 0.5, 1.0 and 2.0 g/kg dose of extract to rats exposed to 10 mg/Kg fine particulate matter (PM_2.5_)	Improved electrocardiogram and decreased cytokine levels in PM_2.5—_exposed rats	[89]
Blueberry/ACNs-rich extract and isolated ACNs	In vitro, 200 μL of single ACN and their corresponding metabolites were added to THP-1 cells at different concentrations (from 0.01 to 10 μg/ mL) and incubated for 24 h.	Mv3glu reduced THP-1 adhesion at all the concentrations with the maximum effect at 10 μg/mL. Cy3glu decreased the adhesion by about 41.8% at 10 μg/mL	[97]
Blueberry/ACNs-rich extract	In vivo, 50, 100 and 200 mg/kg via oral gavage for 5 weeks	Increased the early/late ratio of blood flow across the tricuspid valve and tricuspid annular phase systolic excursion	[106]
Blueberry/ACNs-rich extract	In vivo, the participants were fed with 2 dietary blueberry oral intakes equivalent to ½ and 1 cup/day (75/150 g) for 6 months	Improved endothelial function, systemic arterial stiffness, and attenuated cyclic guanosine monophosphate concentrations	[107]
Cornelian Cherry/ACNs-rich extract	In vivo, the rabbits were fed with 10 and 50 mg/kg bodyweight of cornelian cherry extract	The expression of PPAR-α and PPAR-γ in the aorta was enhanced and was also observed a decrease in triglycerides levels	[108]
Cy supplement	In vivo, 5 mg/kg of body weight orally (gavage) for 5 days and 1-h post-challenge of lipopolysaccharides (LPS)	Cy ameliorated cardiac injury, and cell death, and improved cardiac function	[99]
Cy and Mv	In vitro, 100 µm of ACNs were mixed with lipid vesicles	Cy showed to be a more potent reductant of cytochrome C than ascorbate at the same concentration	[98]
*Hibiscus sabdariffa* aqueous extract	In vivo, the rats were fed with 15, 30 or 60 mg/200 g of bodyweight for 2 weeks	Serum angiotensin-converting enzyme activity and plasma angiotensin II levels were significantly reduced	[109]
Haskap berry/ACNs-rich extract	In vivo, the subjects were fed 100, 200 and 400 mg ACNs for 1 week	Lower diastolic blood pressure and heart rate	[94]
Nuts	In vivo, the participants could choose from eight predefined frequency categories of nut consumption	Nut consumption was inversely associated with the risk of myocardial infarction, heart failure, atrial fibrillation, and abdominal aortic aneurysm in the age-adjusted and sex-adjusted analysis	[93]
Nuts and vegetables	In vivo, the diet was measured using a validated and reliable food frequency questionnaire	Higher intake of nuts and vegetables was inversely associated with serum levels of LDL-C and directly associated with HDL-C levels.	[95]
*Odontonema strictum*/ACNs-rich extract	In vitro, the enriched ACN extract of Odontonema strictum induced vasorelaxant effects in a concentration-dependent manner (10–1000 μg/mL) on mice aortic rings	The contraction of the aortic ring can be blocked since 400 µg/mL of ACNs extract of *Odontonema strictum* inhibited the effects of CaCl_2_ and thromboxane A2 analogue agonist (U46618) in physiological salt solution	[110]
Plum juice/ACNs-rich extract	In vivo, 1 × 300 mL or 3 × 100 mL of plum juice over 3 h on 2 different occasions with a 2-week washout period	A significant reduction in blood pressure and cardiovascular responses were observed	[96]

Legend: ACNs—anthocyanins; Cy3glu—Cyanidin-3-glucoside; Dp—delphinidin; HDL-C—high-density lipoprotein-cholesterol; LDL-C low-density lipoprotein-cholesterol; Mv3glu—malvidin-3-glucoside.

**Table 3:** The effects of ACNs in neurodegenerative diseases.

Source/ACNs	Study Type	Effects and Potential Mechanism	Ref.
*Aronia melanocarpa*/Cy3gal	In vivo, the SAMR1 mice were fed with 25 mg/kg of Cy3gal and with the combination of 100 mg/kg of metformin plus 25 mg/kg Cy3gal	The SAMP8 mice treated with metformin and Cy3gal showed improved spatial learning and memory compared with the SAMP8 model group	[116]
Berry/ACNs-rich extract	In vitro, the BV-2 cells were treated with different concentrations of extract (20, 40, and 80 µg/mL) for 24 h	The berries, including blackberry, black raspberry, blueberry, cranberry, red raspberry, and strawberry, showed free radical scavenging, methylglyoxal-trapping, and anti-glycation effects	[117]
Bilberry/ACNs-rich extract	In vivo, the rats were fed for 12 weeks with ACNs-rich bilberry extract supplementation (0.02%)	Bilberry extract changed the global gene expression profile in the hippocampus of ApoE^−^/^−^ mice. Altered genes are involved in inflammation, neuronal function, synaptic plasticity	[113]
Black beans/ACNs-rich extract	In vivo (APP/PS1 mice) and in vitro (mouse hippocampal HT22 cells) were tested with 100 µg/mL of black bean extract	Regulate the PI3K/Akt/GSK3 pathway and consequently activate the downstream endogenous antioxidant Nrf2/HO-1 pathway and its target genes, reducing the AβO-induced elevation of ROS-mediated oxidative stress and preventing neurodegeneration via a PI3K/Akt/Nrf2-dependent pathway	[118]
Black carrot/ACNs-rich extract	In vitro, SH-SY5Y cells were co-incubated with black carrot extract (2.5, 5, 10, 25, 50, 100 µg/mL) and 0.5 mM 1-methyl-4-phenylpyridinium (MPP+)	Black carrot extract exhibited antioxidant activity via scavenging MPP+-induced ROS and protecting dopaminergic neurons from ROS-mediated apoptosis	[119]
Blueberries/ACNs extract	In vivo, the extracts were incorporated (2% *w/w*) into the standard AIN-76A purified diet for rodents for 6 weeks	The extract improves learning capabilities in aged rats, counteracting spatial memory loss in aged brains, through the modulation of several cell signalling events implicated predominantly in synaptic plasticity, apoptosis, and cytoskeleton remodelling	[115]
Blueberries/ACNs-rich extract	In vitro, N9 cells were pre-treated with the extract for 3 h before the exposure to the stimulus, 1 µg/mL LPS and 0.6 ng/mL IFN-γ	Suppression of NFkB activation, and to a signal transducer and activator of transcription 1-independent Mechanism	[120]
Cy3glu	In vitro, SH-SY5Y cells were co-incubated with Cy3glu (10–100 mM)	Cy3glu increased the expression of nuclear factor erythroid 2-related factor 2, a vital transcription factor for regulating the expression of antioxidant genes, as well as antioxidant enzymes such as superoxide dismutase and catalase	[121]
Cy3glu	In vitro, HMC3 cells were treated with 1 μM of Aβ42 and co-treated with 1 μM of Aβ42 and 50 μM of Cy3glu for 24 h. In vivo, the mice were orally administered 30 mg/kg/day Cy3glu for 38 weeks	Cy3glu was found to upregulate PPARγ expression levels both in vitro and in vivo, whereas a PPARγ antagonist (GW9662) was found to block Cy3glu-mediated effects in vitro	[122]
Cy3glu	In vitro, BV2 cells pretreated with Cy3glu at final concentrations of 2.5, 5, and 10 μM, for 4 h and then stimulated with 1 μg/mL LPS for 24 h	Cy3glu significantly suppresses microglial activation and the production of neurotoxic mediators including nitric oxide, prostaglandin E2, and pro-inflammatory cytokines such as interleukin-1β and interleukin-6 in LPS-activated BV2 cells	[123]
Dp3-gal, Cy3gal, and Mv3gal	In vitro, the disassembly capability of 100 µM of ACNs was evaluated using the macroscopic (conventional) total-internal-reflection-fluorescence microscopy	The disassembly activity to the amyloid β fibrils depends on the number of hydroxyl (OH) groups in six-membered ring B of ACNs, and only Dp3gal, possessing three OH groups there, shows high disassembly activity	[124]
*Lycium ruthenicum* Murr. fruit/Pn3,5-diglu	In vivo, the rats were treated with 100 mg/kg of Pn3,5-diglu by oral gavage after D-galactose administration, once daily for 7 weeks	Pn3,5-diglu alleviate cognitive dysfunction, oxidative stress, neuroinflammation, and shift the abnormal hippocampal metabolites in aging rats induced by D-galactose	[125]
*Hibiscus sabdariffa* L./ACNs-rich extract	In vivo, the mice were tested with a dose of 200 mg/kg of Hibiscus extracts compared with celecoxib (30 mg/kg)	*Hibiscus* was able to reverse the streptozotocin-induced upregulation in the amyloidogenic pathway as well as targeting COX-2/mPGES-1 in PGE2 production and modulating cytokine levels	[126]
*Myrica rubra*/ACNs-rich extract	In vivo, the mice were treated with 100, 150, and 300 mg/kg of Myrica rubra extract every day	The cerebral infarction volume, disease damage, and contents of nitric oxide and malondialdehyde were reduced, while the level of superoxide dismutase was increased in ischemia-reperfusion mice	[127]
Mulberry fruits/Cy3glu	In vivo, the rats were intragastric administrated with 150 mg/kg Cy3glu once daily	Cy3glu may exhibit its neuroprotection via upregulating neurotransmitters levels, protecting the N-methyl-D-aspartic acid receptor function, promoting Ca^2+^ influx and modifying the Ca^2+^ dependent processes like protein kinases, signalling molecules, transcription factors and immediate early genes	[128]
*Nitraria tangutorum* Bobr. fruit/ACNs-rich extract	In vivo, the rats were treated with 50 mg/kg of Nitraria tangutorum Bobr. fruit extract after receiving 100 mg/kg of D-galactose	The extract exhibited neuroprotective effects probably through suppressing oxidative stress, reducing amyloid-beta42 (Aβ42) accumulation, and inhibiting gliosis in the hippocampus of rats	[129]
Purple carrot and flaxseed oil/Mixture of ACNs-rich extract	In vivo, the mice were fed with 100 mg/kg of purple carrot and flaxseed oil mixture of ACNs-rich extract	Significant improvement in acetylcholinesterase, antioxidant enzymes, tumour necrosis factor-α and malondialdehyde in brain tissue and butyrylcholinesterase in plasma	[130]
Strawberries/ACNs-rich extract	In vivo, mice received 2 mg/kg/day of the extract’s primary ACNs constituent for 105 days of the age	The extract significantly reduced astrogliosis in spinal cord and preserved neuromuscular junctions in gastrocnemius muscle.	[131]
Sweet cherry/ACNs-rich extract	In vitro, BV2 microglia and SH-SY5Y neuroblastoma; In vivo, *Drosophila melanogaster* rotenone (ROT)-induced model	25 µg/mL of sweet cherry extract produced a significant increase in the survival rate of nematodes submitted to thermal stress (35 °C, 6–8 h), at the 2nd and 9th day of adulthood	[132]
Wheat grain/ACNs	In vivo, the mice were fed with wheat grain of near isogenic lines differing in ACNs content for 5–6 months	Reduce the alpha-synuclein accumulation and modulated microglial response in the brain of the transgenic mice	[133]

Legend: ACNs—anthocyanins; Cy3gal—cyanidin-3-*O*-galactoside; Cy3glu—Cyanidin-3-glucoside; Dp3gal—delphinidin-3-*O*-galactoside; gal—galactoside; Mv3gal—malvidin-3-galactoside.

## Data Availability

Not applicable.

## Abbreviations

ACNs—anthocyanins; arab—arabinose; BC—breast cancer; BRB—blood retinal barrier; caf—caffeoyl; CC—colon (colorectal) cancers; Cy—cyanidin; Cy3gal—cyanidin-3-*O*-galactoside; Cy3glu—Cyanidin-3-glucoside; Cy3rut—cyanidin-3-*O*-rutinoside; Cy3xyl—Cyanidin-3-xylose; CVDs—cardiovascular diseases; DMH—1,2-dimethylhydrazine; Dp—delphinidin; Dp3gal—delphinidin-3-*O*-galactoside; Dp3glu—delphinidin-3-glucoside; fer—feruloyl; gal—galactoside; glu—glucoside; HDL-C—high-density lipoprotein-cholesterol; LC—lung cancer; LDH—lactate dehydrogenase; LDL-C—low-density lipoprotein-cholesterol; NSCLC—non-small cell lung cancer; Mv—malvidin; Mv3glu—malvidin-3-glucoside; PC -prostate cancer; PD—Programmed cell death protein-1; PD-L1—programmed death-ligand 1; Pg—pelargonidin; Pg3glu- Pelargonidin3-glucoside; phb—*p*-hydroxybenzoyl; Pn—peonidin; Pt—petunidin; ROS—reactive oxygen species; rut—rutinose; sin—sinapoyl; TNBS—2,4,6-trinitrobenzene acid; T2DM—type 2 diabetes mellitus; xyl—xylosyl.
